# Utility and Drawbacks of Chimeric Antigen Receptor T Cell (CAR-T) Therapy in Lung Cancer

**DOI:** 10.3389/fimmu.2022.903562

**Published:** 2022-06-02

**Authors:** Prameela Kandra, Rajender Nandigama, Bastian Eul, Magdalena Huber, Sebastian Kobold, Werner Seeger, Friedrich Grimminger, Rajkumar Savai

**Affiliations:** ^1^ Department of Biotechnology, Gandhi Institute of Technology and Management (GITAM) Institute of Technology, Gandhi Institute of Technology and Management (GITAM) Deemed to be University, Visakhapatnam, India; ^2^ Max Planck Institute for Heart and Lung Research, Member of the German Center for Lung Research Deutsches Zentrum für Lungenforschung (DZL), Member of the Cardio-Pulmonary Institute (CPI), Bad Nauheim, Germany; ^3^ Department of Internal Medicine, Member of the Deutsches Zentrum für Lungenforschung (DZL), Member of Cardio-Pulmonary Institute (CPI), Justus Liebig University, Giessen, Germany; ^4^ Institute for Medical Microbiology and Hygiene, Philipps-University Marburg, Marburg, Germany; ^5^ Division of Clinical Pharmacology, Department of Medicine IV, Member of the Deutsches Zentrum für Lungenforschung (DZL), University Hospital Munich, Munich, Germany; ^6^ German Cancer Consortium Deutsches Konsortium für Translationale Krebsforschung (DKTK), Partner site Munich, Munich, Germany; ^7^ Institute for Lung Health (ILH), Justus Liebig University, Giessen, Germany

**Keywords:** adaptive therapy, CAR-T cells, lung cancer, tumor-associated target antigens, toxicities

## Abstract

The present treatments for lung cancer include surgical resection, radiation, chemotherapy, targeted therapy, and immunotherapy. Despite advances in therapies, the prognosis of lung cancer has not been substantially improved in recent years. Chimeric antigen receptor (CAR)-T cell immunotherapy has attracted growing interest in the treatment of various malignancies. Despite CAR-T cell therapy emerging as a novel potential therapeutic option with promising results in refractory and relapsed leukemia, many challenges limit its therapeutic efficacy in solid tumors including lung cancer. In this landscape, studies have identified several obstacles to the effective use of CAR-T cell therapy including antigen heterogeneity, the immunosuppressive tumor microenvironment, and tumor penetration by CAR-T cells. Here, we review CAR-T cell design; present the results of CAR-T cell therapies in preclinical and clinical studies in lung cancer; describe existing challenges and toxicities; and discuss strategies to improve therapeutic efficacy of CAR-T cells.

## Introduction

Lung cancer is one of the most common and deadly cancer types globally (1). Lung cancer is a highly complex, heterogeneous disease with a poor prognosis. The poor survival rate of patients with lung cancer (5-year survival rate: 10%–20%) is a consequence of advanced stage at presentation (2, 3). Histologically, lung cancer is classified as non-small cell lung carcinoma (NSCLC, approximately 85% of cases) or small cell lung carcinoma (approximately 15% of cases). NSCLC, causing a major proportion of lung cancer-related deaths, is classified as adenocarcinoma, squamous cell carcinoma, or large cell carcinoma (4). Furthermore, genomic profiling studies have uncovered driver mutations in lung cancer that support tumor growth and proliferation. The most frequently found driver mutations in lung cancer are Kirsten rat sarcoma viral (KRAS) oncogene homolog and epidermal growth factor receptor (EGFR) mutations (5).

The present main treatment strategies for lung cancer include surgery, radiotherapy, chemotherapy, targeted therapy, and immunotherapy (6, 7). Although lung cancer is curable when diagnosed at an early stage, it even then remains a challenge due to relapse, and poor survival in >70% of patients (8). Over the past two decades, cytotoxic chemotherapies used to treat lung cancer have evolved to platinum-based chemotherapy, cisplatin-based combination therapies, neoadjuvant therapy, and adjuvant therapy (9). In addition, targeted therapies have also been developed to treat patients with lung cancer harboring EGFR or anaplastic lymphoma kinase mutations (10). Recently, immunotherapy has complemented this arsenal with the discovery and targeting of immune checkpoint inhibitors such as anti-cytotoxic T lymphocyte-associated protein 4 (CTLA-4) and anti-programmed cell death-1 (PD-1) therapies (7). Despite the development of various therapeutic regimens for lung cancer, such therapies only provide durable responses and efficacy in a subset of patients. Variable responses observed under treatment in different tumors might be attributable to disease heterogeneity or tumor heterogeneity across patients. Therefore, it is necessary to explore novel therapies to improve clinical outcomes for more patients. In this setting, next-generation immunotherapeutics, such as immunomodulators and adoptive T-cell therapies including classical T-cell receptor (TCR) and chimeric antigen receptor (CAR)-T-cell therapies, bear promise for treating cancers including lung cancer (11–13).

CAR-T cell therapy has emerged as an innovative cancer immunotherapy for lung cancer treatment (13–16). Although CAR-T cell therapy produced remarkable clinical responses in hematological malignancies (17), this therapy has displayed limited anti-tumor activity in solid tumors including lung cancer. Despite targeting a variety of antigens and tumor types, clinical data for CAR-T cell therapy in solid tumors are disappointing (18). While CAR-T cell therapy has shown clinical success in hematological malignancies, severe toxicities such as cytokine release syndrome (CRS), neurotoxicity, on-target/off-tumor toxicity, tumor lysis syndrome (TLS), and anaphylaxis have also been reported in CAR-T therapy (19). Also, some concerns must be addressed including limited efficacy of CAR-T cell therapies in solid tumors, limited persistence, antigen escape, CAR-T cell trafficking, tumor infiltration, and the immunosuppressive microenvironment (20, 21). Recently, several studies proposed strategies to ameliorate efficacy of CAR-T cell therapy and limit its toxicities (22–24). In this review, we focus on CAR-T cell design, present existing preclinical and clinical studies in lung cancer treatment; highlight existing challenges and toxicities; and also discussed strategies to improve the therapeutic efficacy of CAR-T cells in solid tumors.

## The Design and Structure of CAR-T Cells

**Figure 1:**
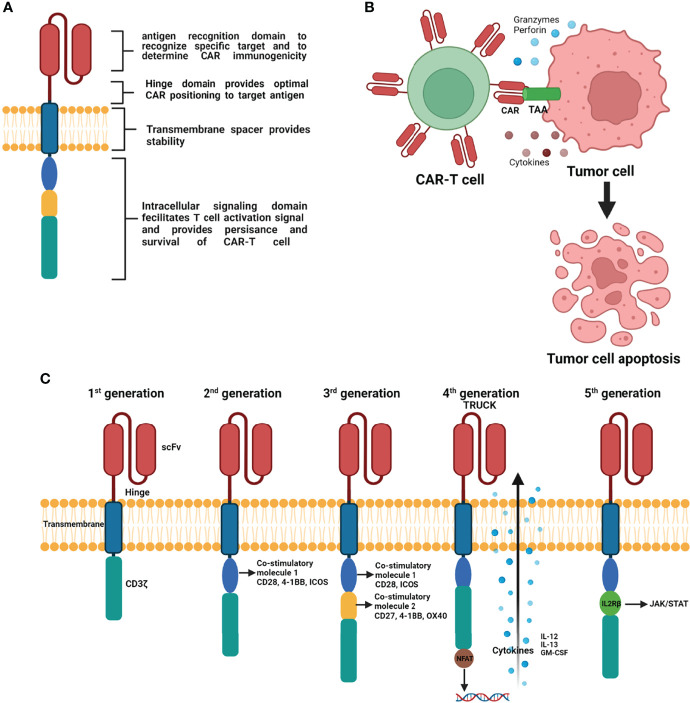
Schematic representation of basic principle of CAR structure **(A)**, mechanism of CAR engineered T-cells action on tumor cells **(B)**, and progressive evolution of CAR-T cells with modifications from 1^st^ generation to 5^th^ generation **(C)** (Figure generated using Bio Render).

T cells genetically engineered to carry synthetic CAR bind specifically targeted tumor antigens and kill these targeted tumor cells. CAR are synthetic receptors composed of an antigen-binding domain/hinge motif, transmembrane domain, and intracellular signaling domain. The extracellular antigen-binding domain, composed of a single-chain variable fragment (scFv), recognizes targeted tumor-associated antigens (TAAs) and triggers downstream signaling. The hinge/spacer region provides flexibility to allow the antigen-binding domain to access the targeted antigen. The hinge/spacer region can be adjusted to its optimal length to provide a sufficient distance between CAR-T cells and targeted tumor cells. The transmembrane domain facilitates the distribution of CARs to the T cell membrane, influencing CAR expression, function, and stability. The intracellular domain or endodomain is composed of combinations of signaling domains such as the T-cell activation complex transducer CD3ζ and several costimulatory molecules (25) (**Figures 1A, B**). The design and structure of CAR have been extensively reviewed elsewhere (26, 27).

To improve the efficacy and safety of CAR-T cell therapy, CAR-T cells have undergone several progressive changes by modifying the CAR structure based on its intracellular signaling domains (**Figure 1C**). The first generation of CAR, containing the antigen recognition extracellular scFv and CD3ζ signaling endodomain, displayed less efficient T cell activation and a short survival time *in vivo* (28–30). To improve the persistence and efficacy of CAR-T cells, second-generation CARs contain an additional costimulatory molecule (e.g., CD28, 41BB, ICOS) that enhances T cell proliferation, prolongs T cell survival time, and improves clinical outcomes (31–33). The design of third generation of CAR included CD3ζ and two costimulatory molecules that further enhance CAR-T cell function. The most commonly used third generation costimulatory molecules are CD27, CD28, 41BB, ICOS, and OX-40 (34, 35). The design of fourth-generation CAR-T cells introduced T cells redirected for universal cytokine-mediated killing containing nuclear factor of activated T cells. These fourth-generation CAR-T cells can produce pro-inflammatory cytokines (interleukin [IL]-12, IL-13, and GM-CSF) upon activation and enhance the penetration ability of T cells to overcome the immunosuppressive effect of the hostile tumor microenvironment (TME) (36). The fifth generation of CAR includes an IL-2Rβ fragment that induces JAK production and activates signal transducer and activator of transcription 3/5 (37).

## CAR-T Cell Therapy Applications and Tumor-Associated Target Antigens in Lung Cancer

**Figure 2:**
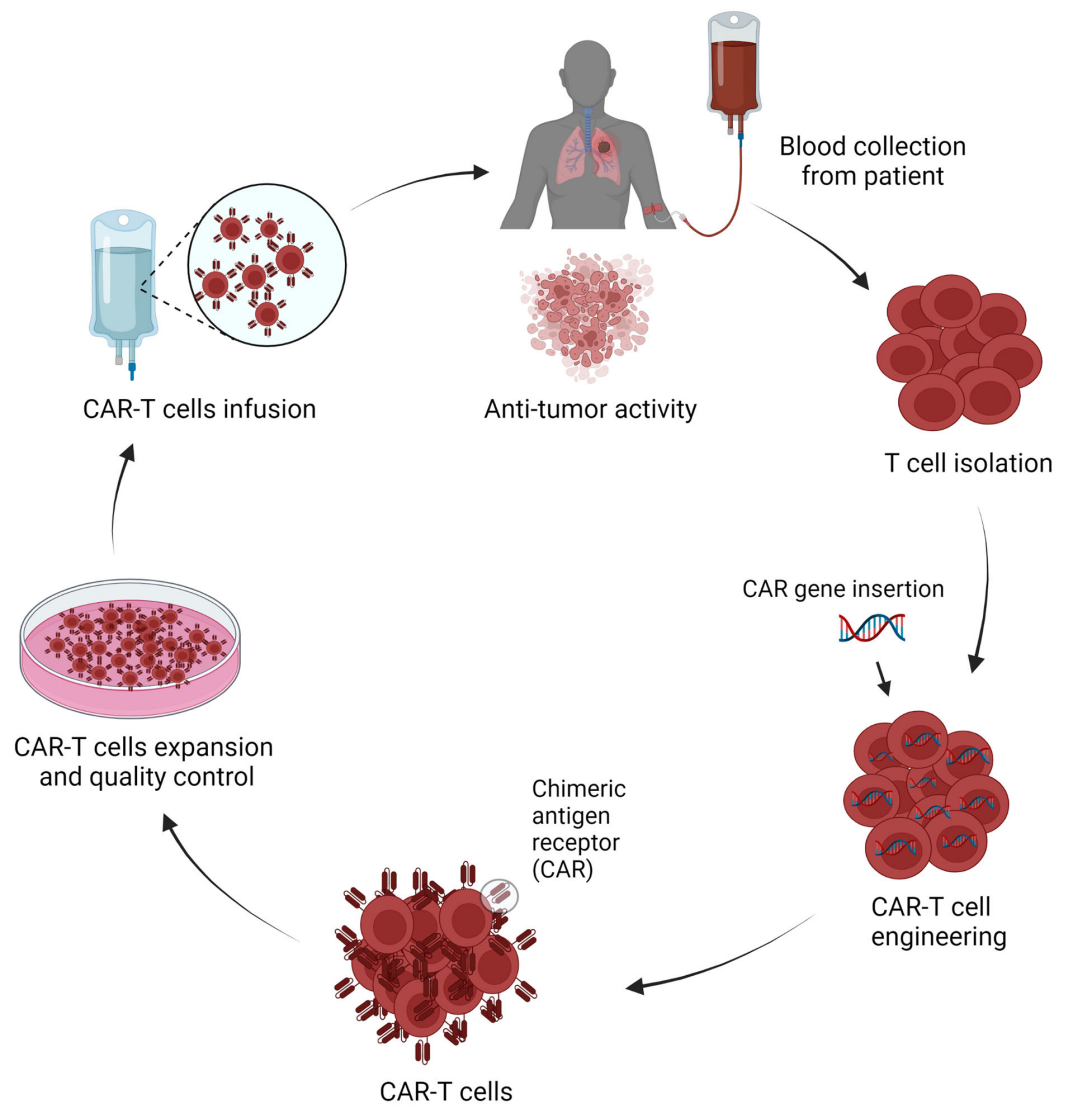
Schematic representation of CAR-T cells production and application in lung cancer treatment (Figure generated using Bio Render).

CAR-T cell therapy is an individualized cell-based therapy that involves the modification of a patient’s own T cells to express CAR. The generation of CAR-T cells involves a complex engineering process featuring several steps starting with the collection of T cells from the patients, engineering cells to express tumor-specific antigen-targeted CAR on their surface, CAR-T cell expansion, and purification, and the infusion of CAR-T cells back into the patient with therapeutic intention (**Figure 2**).

CAR-T cell adaptive cancer immunotherapy has emerged as a promising strategy for the treatment of solid tumors including lung cancer. Synthetic CAR-T cells are independent of major histocompatibility (MHC) complex targeted for TAAs on cancer cells to establish tumor immunity. Similarly, for successful CAR-T therapy in solid tumors, it is important to identify specific TAAs that are highly and selectively expressed in solid tumors but weakly expressed or absent in normal tissue. Several TAAs have been proposed in CAR-T cell research in solid tumors including lung cancer. TAAs currently being investigated in clinical trials of CAR-T cells include carcinoembryonic antigen (CEA), EGFR, human epidermal growth factor receptor 2 (HER2), mesothelin (MSLN), prostate stem cell antigen (PSCA), mucin 1 (MUC1), tyrosine kinase-like orphan receptor 1 (ROR1), programmed death ligand 1 (PD-L1), and CD80/CD86 (**Table 1**).

**Table 1:** Potential TAAs in CAR-T cell therapy clinical tails for lung cancer (ClinicalTrials.gov).

Type of CAR-T	Malignancies	NCT number	Phase	Location	Status
CEA-Targeted CAR-T	Lung, Colorectal, Gastric, Breast, Pancreatic	NCT02349724	I	China	Unknown
CEA-Targeted CAR-T	Lung, Colorectal, Liver, Pancreatic, Gastric, Breast	NCT04348643	I/II	China	Recruiting
CXCR5 modified EGFR-targeted CAR-T	NSCLC	NCT04153799	I	China	Recruiting
CXCR5 modified EGFR-targeted CAR-T	NSCLC	NCT05060796	I	China	Recruiting
EGFR-targeted CAR-T	Advanced solid tumors	NCT01869166	I/II	China	Unknown
HER2-targeted CAR-T	Breast, Gastric, NSCLC	NCT01935843	I/II	China	Unknown
HER2-targeted CAR-T	Breast, Ovarian, Lung, Gastric, Colorectal, Glioma, Pancreatic	NCT02713984	I/II	China	Withdrawn
MSLN-targeted CAR-T	Cervical, Pancreatic, Ovarian, Mesothelioma, Lung cancer	NCT01583686	I/II	United States	Terminated
PD1-MSLN-targeted CAR-T	Advanced solid tumors, Lung Cancer, Mesothelioma	NCT04489862	I	China	Recruiting
Autologous CAR-T cells transfected with anti MSLN mRNA	Mesothelioma	NCT01355965	I	United States	Completed
TnMUC-1-targeted CAR-T	NSCLC, Ovarian cancer, Fallopian tube cancer, Pancreatic ductal adenocarcinoma, multiple myeloma	NCT04025216	I	United States	Recruiting
MUC-1-targeted CAR-T	Lung neoplasm malignant, NSCLC	NCT03525782	I/II	China	Unknown
MUC-1-targeted CAR-T	Hepatocellular carcinoma, NSCLC, Pancreatic carcinoma, breast carcinoma, Ovarian cancer, NSCLC, Colorectal cancer	NCT02587689	I/II	China	Unknown
P-MUC1C-ALLO1-targeted CAR-T	Pancreatic cancer, Renal cell carcinoma, Nasopharyngeal carcinoma, Head and neck squamus cell carcinoma, Gastric cancer	NCT05239143	I	United States	Not yet recruiting
PSCA/MUC1/TGFβ/HER2/Mesothelin/Lewis-Y/GPC3/AXL/EGFR/B7-H3/Claudin 18.2 -CAR-T cell	Advanced lung cancer	NCT03198052	I	China	Recruiting
ROR1-targeted CAR-T	Hematopoietic and Lymphoid Cell Neoplasm, Breast carcinoma, Advanced NSCLC, Chronic lymphocytic leukemia	NCT02706392	I	United States	Recruiting
PD-L1-targeted CAR-T	Advanced Lung cancer	NCT03330834	I	China	Terminated
PD-L1-CD80/CD86-targeted CAR-T	NSCLC	NCT03060343	I	China	Unknown
MAGE-A1, MAGE-A4, MucI, GD2, MSLN-targeted CAR-T	Lung cancer	NCT03356808	I/II	China	Unknown
AMT-253 targeted CAR-T	NSCLC	NCT05117138	II	China	Not yet recruiting
CD276 (B7-H3)-targeted CAR-T	Osteosarcoma, Neuroblastoma, Gastric cancer, Lung cancer	NCT04864821	I	China	Not yet recruiting
CD276 (B7-H3)-targeted CAR-T	Malignant melanoma, Lung cancer, Colorectal cancer	NCT05190185	I	China	Recruiting
GPC3-TGF-β targeted CAR-T	Hepatocellular, Squamous cell carcinoma	NCT03198546	I	China	Recruiting

CEA is a fetal antigen that is expressed during fetal development but is minimally expressed or absent in adult tissues. CEA is overexpressed in various cancers, including 70% of NSCLC (38). Therefore, CEA has proven useful as a tumor marker and for monitoring the response to CEA-targeted CAR-T therapy. Furthermore, preclinical studies also showed the relevance of serum CEA concentrations as an indicator of brain metastases in patients with advanced NSCLC (39). This led to the establishment of CEA-targeted CAR-T cells in phase I clinical trials to evaluate the efficacy, safety, and maximum tolerated dose of this therapy in various solid tumors including lung cancer (NCT02349724, NCT04348643). In addition, *in vivo* established human lung cancer model in immune-compromised mice showed treatment with inducible IL8 (iIL8) and CEA-targeted CAR-T cells completely eliminated advanced stage of lung cancer (40).

EGFR, expressed in both epithelial cells and epithelium-derived malignancies, is a transmembrane glycoprotein belonging to the tyrosine kinase receptor family. In addition to EGFR overexpression in solid tumors including NSCLC, it has also been reported that more than 60% of EGFR mutations are associated in NSCLC patients (41). Therefore, EGFR has become a possible therapeutic target in CAR-T cell therapy for NSCLC. *In vitro* studies revealed that EGFR-targeted CAR-T cells exhibit specific cytolytic activity and produce high levels of cytokine (IL-2, IL-4, IL-10, TNF-α, and interferon-γ [IFN-γ]) against EGFR-positive tumor cells (42). There are two ongoing phase I clinical trials in lung cancer of C-X-C chemokine receptor type 5 modified EGFR-targeted CAR-T cells (NCT04153799, NCT05060796). Furthermore, phase I/II clinical studies in patients with advanced NSCLC revealed no severe toxicity after 3–5 days of EGFR-targeted CAR-T cell perfusion (NCT01869166). These studies indicate the promise of EGFR-targeted CAR-T cells in treating NSCLC.

HER2, a member of the tyrosine kinase erythroblastic leukemia viral oncogene homolog (ERBB) family, is also a potential CAR target antigen in lung cancer (43). Studies using an *in vivo* A549 NSCLC xenograft model and *in vitro* NSCLC cell lines (A549 and H1650) revealed anti-tumor effects of HER2-targeted CAR-T cells, including decreased tumor growth but not complete tumor elimination (44, 45). In addition, two phase I/II clinical studies of HER2-targeted CAR-T cells in treating NSCLC have been launched (NCT01935843, NCT02713984). However, clinical data have not yet been reported for HER2-targeted CAR-T cell therapy in NSCLC.

MSLN, a cell surfaced glycoprotein, is overexpressed in the majority of cancer types including lung cancer, mesothelioma, pancreatic cancer, and ovarian cancer (46, 47). High expression of MSLN occurs in approximately 69% of lung adenocarcinomas, and it carries an increased risk of recurrence with reduced overall survival in NSCLC (48, 49); therefore, it could be a potential target in CAR-T cell therapy. This prompted the development of MSLN-targeted CAR-T cells, and research using *in vivo* subcutaneous mouse lung cancer models and *ex vivo* models revealed a slower tumor growth rate and inhibitory effects on cell proliferation (46, 50). However, phase I/II clinical trials of MSLN-targeted CAR-T cells in MSLN-positive metastatic lung cancer were terminated because of poor accrual (NCT01583686). Furthermore, the intravenous application of mRNA-engineered T-cells expressing MSLN-targeted CARs did not exert effects on metastatic tumors in patients with NSCLC (NCT01355965).

MUC1 is another potential candidate that is aberrantly overexpressed in NSCLC and other cancer types (51). MUC1 is an abnormally glycosylated extracellular transmembrane glycoprotein that is correlated with poor survival and tumor progression (52). Ongoing phase I clinical trial studies are examining Tn glycoform of MUC1-targeted CAR-T cells for the treatment in MUC-1 positive advanced cancers, including NSCLC (NCT04025216). Additionally, phase I/II clinical studies in various solid tumors including lung cancer using MUC-1–targeted CAR-T cells have been launched (NCT03525782, NCT02587689). Meanwhile, an early stage clinical trial is using P-MUC1-ALLO1–targeted CAR-T cells in solid tumors including lung cancer is ongoing (NCT05239143). In contrast, studies using MUC-1–targeted CAR-T cells in patient xenograft model did not reveal significant suppression of NSCLC tumor growth (51).

PSCA is a glycophosphatidylinositol-anchored cell surface protein that is aberrantly overexpressed in NSCLC (51). Using *in vivo* PDX subcutaneous mouse models and *in vitro* models, the combination of CAR-T cells targeting MUC-1 and PSCA substantially inhibited tumor growth and PSCA- and MUC-1–expressing NSCLC cell proliferation (51). Meanwhile, an ongoing phase I study is testing safety, efficacy, and tolerance of a combination of CAR-T cells targeting MUC-1 and PSCA in lung cancer (NCT03198052).

ROR1, a tyrosine kinase-like orphan receptor, is highly expressed in NSCLC, breast cancer, and other solid tumors (53, 54). Because of the toxicity of ROR1-targeted CAR-T cells attributable to ROR1 expression in normal tissues, CAR-T cells have been engineered with synthetic Notch receptors EpCAM and B7-H3 to improve selectivity, specificity, and tumor regression in ROR1-expressing tumor cells with less toxicity (55). A phase I clinical study was designed to assess the safety and anti-tumor effects of ROR1-targeted CAR-T cells in ROR-positive NSCLC (NCT02706392). In addition, animal models examining ROR1-targeted CAR-T cells revealed effective elimination of ROR1-positive NSCLC cells (53).

Treatments targeting the PD-1-PD-L1 complex, which blocks the cytotoxic T-cell activity, have made substantial progress in NSCLC and other cancer types (56, 57). *In vitro* and *in vivo* studies using PD-L1–targeted CAR-T cells revealed cytotoxic effects and tumor growth inhibition in NSCLC cells (58, 59). However, phase-1 clinical trials of PD-L1 targeted CAR-T cells in advanced lung cancer patients were terminated because of serious adverse effects (NCT03330834). Also, another phase I clinical trial has been ongoing with PD-L1-MSLN targeted CAR-T cells to determine safety and efficacy in PD-L1–positive NSCLC patients (NCT04489862).

The expression of CD80/CD86, costimulatory molecules of the immune system, has been detected in NSCLC (60). CD80 and CD86 bind to CTLA-4 and downregulate T-cell function, making them preferred targets for immune intervention (61). Phase I clinical trial study is ongoing to assess safety and tolerance of PD-L1 and CD80/CD86 targeting CAR-T cells in the treatment of recurrent or refractory NSCLC patients (NCT03060343). In addition, CD80/CD86-targeted CAR-T cell treatment controlled tumors including NSCLC tumors by reversing inhibitory CTLA-4–CD80/CD86 signals (62).

Fibroblast activator protein (FAP), highly expressed in cancer-associated fibroblasts (CAFs), can modulate the tumor microenvironment by ECM remodeling. FAP overexpression on CAFs is associated with poor prognosis in many solid tumors including lung cancer. Targeting FAP is also being evaluated for CAR-T cell therapy in NSCLC. *In vitro* studies in A549 cells using FAP targeted CAR-T cells showed significant reduction of tumor growth (63, 64). Furthermore, mouse model studies using FAP-targeted CAR-T cells showed 35-50% reduction of tumor growth after treatment (63, 64).

Preclinical CAR-T cell therapy studies in lung cancer by targeting several potential targets, like erythropoietin-producing hepatocellular carcinoma A2 (EphA2), lung-specific X protein (LUNX), variant domain 6 of CD44 gene (CD44V6), melanoma-associated antigen (MAGE)-A1, exhibited significant suppression of tumor growth (65–68). Furthermore, potential targets like MAGE-A1 (NCT03198052 and NCT03356808), AMT-253 (NCT05117138), CD276 [(B7-H3): NCT04864821, NCT05190185), and GPC3-transforming growth factor beta (TGF-β; NCT03198546), are under evaluation for CAR-T cell therapy application in NSCLC in clinical trials.

## Current Challenges and Toxicities in CAR-T Cell Therapy

Although there has been continuous improvement of CAR-T cell therapies and their great promise in the treatment of lung cancer and other solid tumors has been revealed, many challenges and hurdles exist. T cell intrinsic as well as tumor-driven mechanisms and treatment-related toxicities in CAR-T cell limit efficacy and safety in solid tumors including lung cancer.

### Challenges in Applying CAR-T Cell Therapy in Lung Cancer

**Figure 3:**
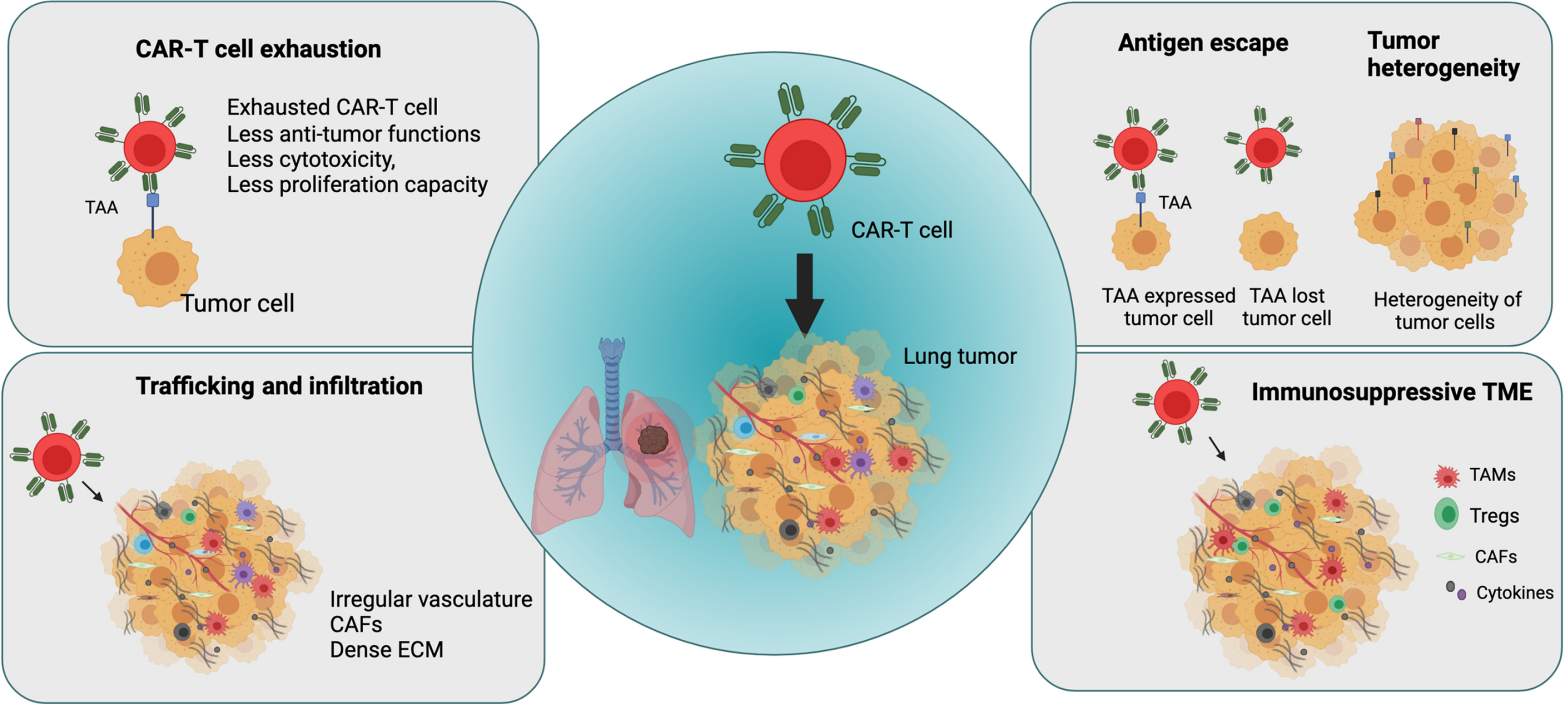
Major challenges in applying CAR-T cell therapy in lung cancer (Figure generated using Bio Render).

Following administration, CAR-T cells encounter considerable challenges in treating lung cancer, such as tumor antigen escape, TME heterogeneity, immune suppression, CAR-T cell trafficking and infiltration into the tumor, and CAR-T cell exhaustion (**Figure 3**). In this section, we further elaborated explaining main current challenges in CAR-T cell therapies in lung cancer and other solid tumors.

The challenges associated with CAR-T cell therapy in solid tumors such as lung cancer include tumor antigen escape and the emergence of multiple resistance mechanisms. Although CAR-T cell therapy can produce high initial response rate in some patients or diseases by overcoming HLA restriction and MHC I downregulation, many patients subsequently experience disease relapse because of antigen escape by cancer cells, resulting in the partial or complete loss of target antigen expression. CD19-targeted CAR-T cell therapy in ALL patients and BMCA-targeted CAR-T cell treatment in patients with multiple myeloma resulted in disease recurrence with the development of resistance and reduced target antigen expression in cancer cells after treatment (69, 70). Similarly, treatment with IL-13Ra2–targeted CAR-T cells in glioblastoma resulted in relapse because of reduced IL-13Ra2 expression in tumors (71). Therefore, it is important to optimize target antigen selection to prevent antigen escape mechanisms, thereby improving anti-tumoral effects of CAR-T cells and preventing disease relapse.

Another significant limiting factor in CAR-T cell therapy in solid tumors including lung cancer is tumor heterogeneity. Overall, tumor heterogeneity is a major factor in cancer treatment efficacy, resistance, and failure (71, 72). Spatial distribution studies in patients with NSCLC revealed high spatial heterogeneity of the intratumoral microenvironment in lung tumors for immune and stromal cells and their impact on survival in lung cancer (73). For example, the heterogeneity of PD-L1 expression in the TME influences the prognosis of lung cancer and significantly affects immunotherapy outcomes (74). It is important to optimize the selection of tumor-specific antigens that are specifically expressed in tumor cells to increase anti-tumor activity and safety of CAR-T cells (75). However, it is highly challenging to identify specific target antigens that are expressed homogenously and stably on tumor cells but not healthy cells.

Similarly, as other solid tumors, CAR-T cells in lung cancer suffer the immunosuppressive effect of the TME, which hinders their effector function and impedes clinical efficacy of CAR-T cells (75, 76).

Many infiltrating cell types contribute to an immunosuppressive TME, including myeloid-derived suppressor cells, CAFs, tumor-associated macrophages, and regulatory T cells, which secrete factors such as TGF-β, IL-10, ARG-1, inducible nitric oxide synthase (iNOS), COX2, PGE2, FAP, and PD-L1 (77, 78). These factors regulate metabolism, cytokine networks, and immune checkpoints in the TME and generate an immunosuppressive microenvironment, thereby leading to reduction or loss of CAR-T cell function.

Unlike observations in hematological malignancies, hurdles including effective trafficking and infiltration of CAR-T cells into the tumor site limit efficacy of CAR-T cell therapy in solid tumors such as lung cancer. T cell infiltration into lung tumors is mainly influenced by chemokines, chemokine receptors, adhesion molecules, the irregular and extensive leakage of the tumor vasculature, and a hypoxic and immunosuppressive TME (79–82). Furthermore, CAFs and extracellular matrix (ECM) establish a physical barrier that causes therapeutic resistance and blocks the penetration of drugs into solid tumors. In lung cancer, the dense fibrotic environment generated by abnormally dense collagen, ECM deposition, and CAF activation impedes immune cell infiltration and the efficacy of immunotherapy (83, 84). Several of these factors and physical barriers in the TME in solid tumors including lung tumors represent the first obstacles encountered by CAR-T cells after administration, thereby impeding trafficking and tumor penetration.

The success of CAR-T cell therapy is also hampered by the development of a dysfunctional state called CAR-T cell exhaustion. CAR-T cell exhaustion is one factor limiting the efficacy of CAR-T cell therapy in solid tumors including lung cancer. T cell exhaustion develops in the TME by persistent antigen stimulation, increase in expression of inhibitory receptors, and the presence of inhibitory immune cells and cytokines (79). In solid tumors, the NR4A transcription factor family plays an important role in T-cell exhaustion, which limits CAR-T cell function in solid tumors (85).

### CAR-T Cell Treatment Related Toxicities

**Figure 4:**
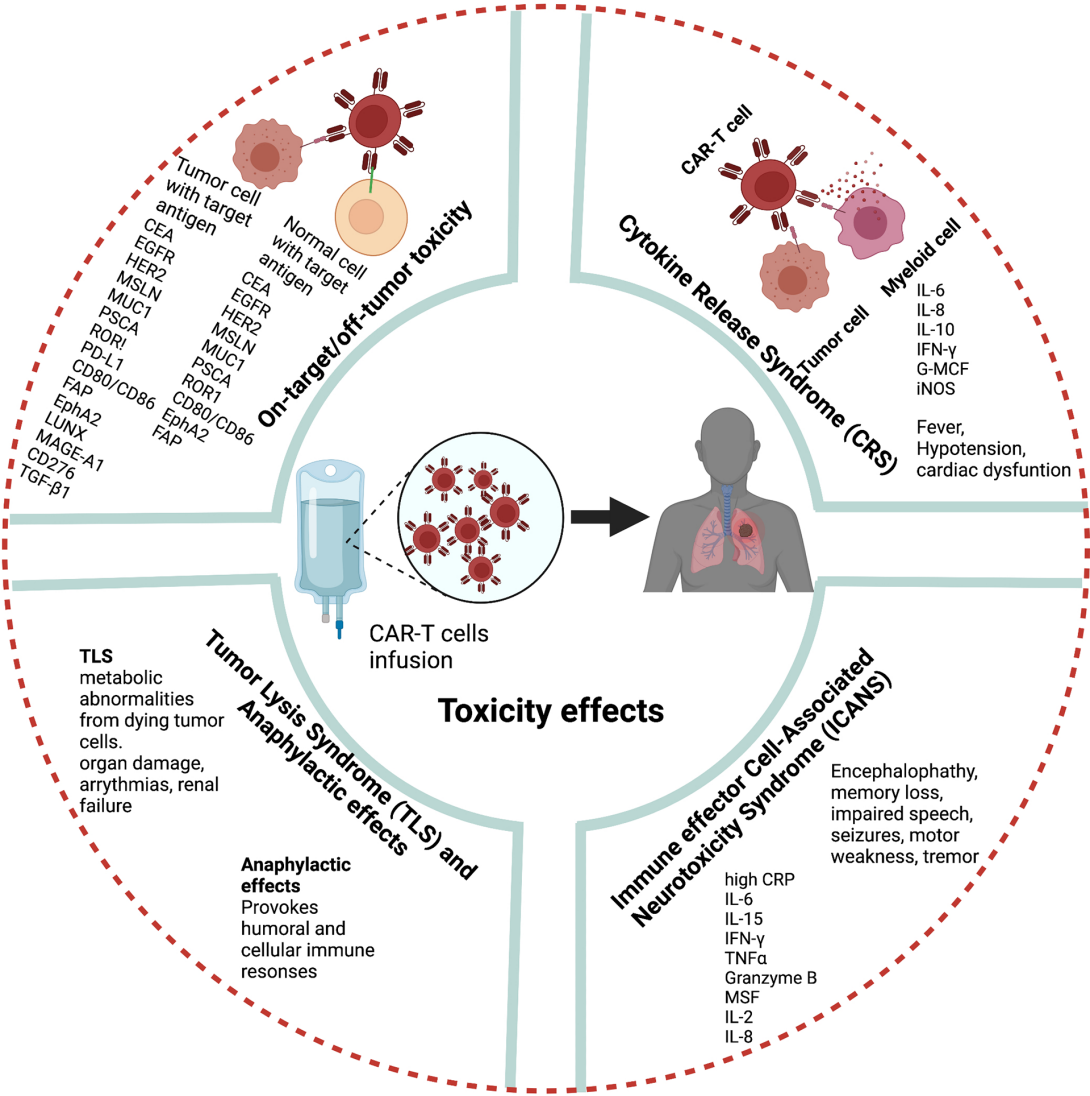
Toxicity hurdles in CAR-T cell therapy (Figure generated using Bio Render).

A major hurdle to CAR-T cell therapy is severe toxicities. The most common toxicities following infusion of CAR-T cells are CRS, neurologic toxicity, tumor lysis syndrome (TLS), on-target-off-tumor effects, anaphylaxis, and hematologic toxicities (19, 86, 87) (**Figure 4**). However, these toxicities are mainly based on clinical observations in hematological malignancies, and the toxic effects and risks of CAR-T cell therapies in lung cancer and other solid tumors must be carefully weighed to expand their clinical use.

CRS is the most prevalent adverse effect after CAR-T cell therapy. Fever is the most common symptom of CRS after CAR-T cell infusion can be accompanied by nausea, fatigue, hypotension, and cardiac dysfunction (88). After CAR-T cell therapy, high IL-6 levels in patient serum are strongly correlated with CRS severity (89). The pathophysiology of CRS related to CAR-T cell therapy is associated with the activation and proliferation of CAR-T cells and release of high levels of several cytokines and chemokines including IFN-γ, IL-6, IL-8, IL-10, granulocyte macrophage colony-stimulating factor, and iNOS which in turn activate endogeneous myeloid cells (90–92).

Neurologic toxicity is the second major side effect reported in patients after CD19-specific CAR-T cell infusion (93). More recently, a CAR-T cell-related encephalopathy syndrome termed immune effector cell-associated neurotoxicity syndrome (ICANS) has been reported (94). The clinical features of ICANS associated with CAR-T cell therapy include encephalopathy, memory loss, seizures, impaired speech, tremor, headache, language disturbance, and motor weakness (95, 96). Although the pathogenesis of ICANS is less clear than that of CRS in CAR-T cell therapy studies, high levels of C-reactive protein, IL-6, IL-15, IFN-γ, TNF-α, granzyme B, granulocyte macrophage colony-stimulating factor, IL-2, and IL-8 are associated with severe ICANS (95, 97, 98). Recently, several studies suggested that blood–brain barrier dysfunction is the main factor in the pathogenesis of neurotoxicity after CAR-T cell therapy (95).

Another potential adverse effect of CAR-T cell therapy is TLS. TLS describes a group of metabolic abnormalities that may occur because of the CAR-T cell-mediated lysis of malignant cells (99). TLS can lead to organ damage, life-threatening arrhythmias, and renal failure.

Further treatment-related side effects may occur if target antigens selected for CAR T cell therapy are not specific and shared healthy tissue and healthy cells. Recent single cell analysis and other studies in healthy tissues studies revealed expression of several TAAs in various non-cancerous tissues, supporting the concern for on-target – off tumor mediated side effects (100–102) (**Table 2**). On-target-off-tumor toxicity through damage in noncancerous normal tissues lead to life-threatening effects (103). Carbonic anhydrase IX-specific CAR-T cell therapy in renal carcinoma resulted in on-target-off-tumor toxicity in the bile duct epithelium and cholestasis because of the expression of the same antigen (29, 104).. Furthermore, CAR-T cell therapy using HER2/neu-specific CAR-T cells resulted in on-target-off-tumor toxicity, leading to respiratory failure, multi-organ dysfunction, and subsequent death because of antigen recognition on pulmonary tissue (105). Dramatic effects have been noted in genetically modified TCRs against melanoma through lethal cardiac toxicity attributable to off-target reactivity (106). CAR-T cell therapy in metastatic colon cancer induced adverse effects within 15 min as a consequence of respiratory dysfunction (105).

**Table 2:** Expression of TAAs in healthy tissues.

Target antigen in CAR-T	Expression in normal tissues
EGFR	Fibroblasts, smooth muscle cells, pericytes, alveolar type1 and type2, ciliated cells, basal cellsrenal epithelium, liver epithelial cells, various pancreatic cell populations
MUC1	alveolar type1 and type2, ciliated cells, basal cells, collecting duct principal cells, liver epithelial cells,ductal cells
MSLN	alveolar type1 and type2, ciliated cells, basal cells, collecting duct principal cells, ductal cells
Her2 (ERBB2)	Fibroblasts, smooth muscle cells, pericytes, alveolar type1 and type2, ciliated cells, basal cells, renal epithelium, liver epithelial cells, various pancreatic cell populations, bone marrow cells CD8 cells, NK cells
FAP	Fibroblasts, smooth muscle cells, pericytes
ROR1	Fibroblasts, smooth muscle cells, pericytes, renal epithelium
EphA2	Cardiac endothelial cells, fibroblasts, smooth muscle cells, pericytes, alveolar type1 and type2, ciliated cells, basal cells, liver epithelial cells
CEA	Colon epithelium
PSCA	Subset of basal and secretary cells of healthy prostrate, pancreatic islets
CD80/CD86	Dendritic cells, macrophages and B cells

As the majority of currently utilized CAR-T cells carry an antigen recognition domain derived from murine monoclonal antibodies (103), infusion may provoke humoral and cellular immune responses culminating in anaphylactic reactions (28, 107). A clinical trial identified cardiorespiratory failure after a third infusion of MSLN-targeted CAR-T cells as a consequence of such species mismatch. Furthermore, this study reported an IgE-mediated anaphylactic event caused by the presence of human anti-mouse antibodies and elevated trypsin antibodies in patient serum (108). These adverse effects might be related to isotype switching to IgE; inappropriate timing of treatment, and improper treatment intervals.

## Future Strategies to Improve CAR-T Cell Therapy

Despite the success of CAR-T cell therapy against hematologic malignancies, the effects of CAR-T cell therapies on solid tumors such as lung cancer are unsatisfactory because of antigen heterogeneity, an immunosuppressive microenvironment, and insufficient trafficking to tumor tissue. Furthermore, CAR-T therapy in the treatment of solid tumors may result in adverse cytotoxicity in healthy cells because of the presence of targeted TAA on healthy cells. Therefore, it is utmost important to develop strategies to improve safety and efficacy of CAR-T cell therapies in lung cancer and other solid tumors. To overcome these hurdles, several studies adapted genetic engineering approaches to modulate CAR-T cells to enhance their efficacy, functional activity in the immunosuppressive TME, and efficient infiltration into the tumor site.

### Modulating CAR Activity

Recently, several scientists attempted to improve the efficacy and feasibility of CAR-T cell therapy in solid tumors and avoid off-tumor toxicity. To overcome antigen heterogeneity in solid tumors, several approaches have been adopted to target multiple antigens with a single CAR-T cell population. The combination of biotinylated antibodies and avidin-conjugated CAR has been used to control CAR-T cell activity and target multiple antigens (109, 110). Other CAR that can potentially target multiple antigens include split universal and programmable (SUPRA) CAR and leucine-zipper motif CAR (ZipCAR) with free scFv motifs (ZipFv). SUPRA CAR reduce CAR-T cell hyperactivity, overcome tumor immune escape, and enhance the activation of T-cells with high sensitivity for various tumor antigens (111). SUPRA CAR also regulate various signaling pathways in T-cells and other cells and prevent CRS. ZipCAR with different types of ZipFv motifs can be designed to recognize various tumor antigens and attenuate the unspecific activation of CAR-T cells. Further strategies using tandem CAR-T cells and dual CAR-T cells that prevent on-target/off-tumor toxicity by targeting two different tumor surface antigens and enhance anti-tumor activity have been reported (112, 113). Modular CAR approaches have been extensively reviewed elsewhere (114).

### Small Molecules-Based or Chemogenitic-Based Switchable CAR-T Cells

To mitigate CAR-T cells posed challenges and complications, further approaches such as small molecules-based or chemogenitic-based switchable CAR-T cells have been developed to regulate CAR activity. A variety of small molecules such as FITC-conjugated antibodies, rapamycin, folate, rimiducid, proteolysis-targeting chimera (PROTAC), and dastinib have been employed to develop safety switches for CAR-T cells (115) Switchable CAR-Ts approach in breast cancer treatment using Her2-targeted antibody drug combination with a T cell-redirected bsAb, and a FITC-modified antibody capable of redirecting anti-FITC CAR-T (switchable CAR-T; sCAR-T) cells showed improved activity against cancer cells (116). *In vitro* and *in vivo* studies using chemically programmed antibody fragment (ca-Fab)/CAR-system based on/off switch targeting folate binding proteins showed specific elimination of folate receptor expressing cancer cells (117). In addition, recent study developed chemogenitic-based switchable CAR-T cells targeting CD19 positive cancer cells in *in vitro* and *in vivo* using anti-CD 19 hepatitis C virus NS3 protease (HSV-NS3) between the single-chain variable fragment (scFV) demonstrated control of CAR-T activity in the presence and absence of HCV-NS3 inhibitor asunaprevir in eliminating CD19 positive tumor cells (118).

### Enhancing CAR-T Cell Therapy to Overcome an Immunosuppressive TME

To improve efficacy of CAR -T-cell therapy, several strategies modified CAR-T cells to secrete pro-inflammatory cytokines such as IL-12 (119) or transgenically express cytokines such as IL-23, IL-12, and IL-15 to protect CAR-T cells in the inhibitory TME and thereby improve their anti-tumor activity (120–124). To reduce cytokine secretion in CAR-T cell therapy, internal ribosome entry site-based approaches can be used in CAR-T cell construction when a cytokine gene is placed 3’ prime of internal ribosome entry site (125). Another study suggested that constructing a constitutive signaling of cytokine receptor C7R, which potentially triggers IL7 stimulation, increase CAR-T cell persistence and antitumor activity (126). Another approach to target PD-1-PD-L1 interaction is programming CAR-T cells to secrete blocking agents for checkpoint inhibitor PD-1. CAR-T cells secreting scFv targeting PD-1 provided a better outcome in PD-L1–positive xenograft mouse models (127). Also, to overcome an immunosuppressive TME, several studies suggested that the combination of monoclonal antibodies inhibiting immune checkpoints such as PD-1 or CTLA-4 and CAR-T cell therapy might result in improved anti-tumor activity (128, 129). In addition, several other approaches developed CRISPR/Cas9-mediated PD1-disrupted CAR-T cells and CTLA-4–specific CAR-T cells to improve effector function of CAR-T cells and enhance their anti-tumor activity (130). In addition to CRISPR/Cas9, several gene-editing tools including zinc finger nucleases, mega nucleases, and transcription activator-like effector nucleases have been applied to engineer CAR-T cells (131, 132).

### Enhancement of Infiltration of CAR-T Cells Into Solid Tumors

To enhance the penetration of CAR-T cells into solid tumors by overcoming physical barriers in the TME, different approaches have been explored to design CAR-T cells targeting the tumor-associated stromal fibroblast protease FAP or ECM-modifying enzymes or to use distinct chemokine gradients to recruit CAR-T cells to solid tumor tissues. Several studies also reported that solid tumor-associated chemokine release characteristics can be utilized to enhance the trafficking of therapeutic T-cells using chemokine receptors (133–135). One study of FAP-targeted CARs in immunocompetent models reported bone toxicity in FAP-positive stromal cells in bone marrow, whereas another study observed reduced tumor growth without toxicities (136). Thus, FAP-targeted CAR-T cell strategies require further deep investigation to explore their efficacy and toxicity. Another approach to enable the expression of heparanase in ECM is targeting heparin sulfate proteoglycans by combining this enzyme with anti-GD2 CAR-T cells. This approach resulted in the increased infiltration of CAR-T cells and prolonged survival in a mouse xenograft tumor model (137). However, these approaches require further research because of the complicated and unpredictable effects of ECM-modifying enzymes.

### Improving Metabolic Functions of CAR-T Cells in TME

Nutrient depletion, hypoxia and toxic metabolites in TME affects biological properties of infiltrating immune cells in solid tumors. These toxic metabolites harbors reactive oxygen species in TME and thereby impairs T cell function (138–140). The efficacy of CAR-T therapy is closely associated with T cell metabolism fitness. Several strategies have been explored to modulate metabolic function of adoptively transferred CAR-T cells including manipulating ROS levels balance, relieving unfavorable metabolic TME, and blocking inhibitory effects of toxic metabolites. To protect CAR-T cell from ROS damage, investigators developed genetically modified T cells which secrete ROS scavenger catalase (141). In order to improve arginine re-synthesis in adoptively transferred T cells, several studies developed either *ex vivo* loading of CAR-T cells with arginine (142) or genetic manipulation of CAR-T cells with arginine synthesizing enzymes to re-synthesize arginine (143). In addition, several other approaches also explored to manipulate glutamine metabolism in the TME to increase T cell effector function (144, 145). Potential strategies to modulate metabolic properties of CAR-T cells have been extensively reviewed elsewhere (146).

### Combinatorial Therapy Approaches

In order to enhance effector function of CAR-T cells, numerous studies are approaching CAR-T cell therapy by combining other therapeutic methods to improve outcomes. These CAR-T cell combinatorial therapies which are being pursued include chemotherapy, radiotherapy, cytokine therapies, checkpoint blockades, and oncolytic viruses (147–150). Combining checkpoint blockade and CAR-T cell therapy may produce a synergic effect and provide infiltration of immune cells into tumors (151).

## Conclusion

Over the last decade, CAR-T cell therapy has revolutionized the treatment of hematological malignancies. The clinical application of CAR-T cell therapy and the identification of novel potential target antigens in lung cancer are the subjects of ongoing research. However, the successful use of CAR-T cell therapy against solid tumors including lung cancer is hampered by several hurdles including antigen targeting, tumor heterogeneity, the immunosuppressive TME, CAR-T cell trafficking, associated toxicities, and on-target-off-tumor effects. Several new strategies are being developed to overcome these obstacles and improve the efficacy and scope of CAR-T cell therapies to permit their more widespread use in cancer treatment. In summary, novel strategies of CAR-T cell design with reduced toxicity that efficiently direct CAR-T cells to tumors may provide a path for their safer and more effective use against different cancer types including lung cancer.

## Author Contributions

All authors listed have made a substantial, direct, and intellectual contribution to the work and approved it for publication.

## Funding

This work was supported by the Max Planck Society, Cardio-Pulmonary Institute (CPI), the German Center for Lung Research (DZL) and DFG, SFB 1213 (Project A10* to RS). This study also supported by the international doctoral program “i-Target: immunotargeting of cancer” (funded by the Elite Network of Bavaria), Melanoma Research Alliance (grant number 409510 to SK), Marie Sklodowaska-Curie Training Netwrok for Optimizing Adoptive T cell Therapy of Cancer (funded by the Horizon 2020 Program of the European Union: grant 955575), Else Kröner Fresenius-Stiftung, German Cancer Aid, Ernst Jung Stiftung, Institutional Strategy LMU excellent of LMU Munich (within the framework of the German Excellence Intiative), Bunderministerium für Bildung Und Forschung, European Research Council (Starting Grant 756017), Deutsche Forschungsgemeinschaft (DFG), by the SFB-TRR 338/1 2021-452881907, FritzBender Foundation, Jose´Carreras Foundation and Hector Foundation.

## Conflict of Interest

SK has received honoraria from TCR2 Inc, Novartis, BMS and GSK. SK is an inventor of several patents in the field of immune-oncology. SK received license fees from TCR2 Inc and Carina Biotech. SK received research support from TCR2 Inc. and Arcus Bioscience for work unrelated to the manuscript.

The remaining authors declare that the research was conducted in the absence of any commercial or financial relationships that could be construed as a potential conflict of interest.

## Publisher’s Note

All claims expressed in this article are solely those of the authors and do not necessarily represent those of their affiliated organizations, or those of the publisher, the editors and the reviewers. Any product that may be evaluated in this article, or claim that may be made by its manufacturer, is not guaranteed or endorsed by the publisher.

## Abbreviations

ALK, Anaplastic lymphoma kinase; CAF, Cancer-associated fibroblasts; CAR, Chimeric antigen receptor; CEA, Carcinoembryonic antigen; CRS, Cytokine release syndrome; EGFR, Epidermal growth factor receptor; FAP, Fibroblast activator protein; ICANS, Immune effector cell-associated neurotoxicity syndrome; KRAS, Kirsten rat sarcoma; NFAT, Nuclear factor of activated T-cells; NSCLC, Non-small cell lung carcinoma; PSCA, Prostate stem cell antigen; SB, Sleeping beauty; TAA, Tumor-associated antigens; TALEN, Transcription activator-like effector nucleases; TCR, T-cell receptor; TLS, Tumor lysis syndrome;
